# The Effects of Caffeine Ingestion on Measures of Rowing Performance: A Systematic Review and Meta-Analysis

**DOI:** 10.3390/nu12020434

**Published:** 2020-02-08

**Authors:** Jozo Grgic, Francisco Javier Diaz-Lara, Juan Del Coso, Michael J. Duncan, Jason Tallis, Craig Pickering, Brad J. Schoenfeld, Pavle Mikulic

**Affiliations:** 1 Institute for Health and Sport (IHES), Victoria University, Melbourne, 3011, Australia; Francisco.DiazLara@vu.edu.au; 2 Centre for Sport Studies, Rey Juan Carlos University, Fuenlabrada, 28943 Madrid, Spain; juan.delcoso@urjc.es; 3 Centre for Sport, Exercise and Life Sciences, Alison Gingell Building, Coventry University, Priory Street, Coventry CV1 5FB, UK; aa8396@coventry.ac.uk (M.J.D.); ab0289@coventry.ac.uk (J.T.); 4 Institute of Coaching and Performance, School of Sport and Wellbeing, University of Central Lancashire, Preston PR1 2HE, UK; craigpickering1014@hotmail.com; 5 Department of Health Sciences, Lehman College, Bronx, NY 10468, USA; bradschoenfeldphd@gmail.com; 6 Faculty of Kinesiology, University of Zagreb, Zagreb 10000, Croatia; pavle.mikulic@kif.hr

**Keywords:** caffeine, ergogenic aid, performance-enhancing effect

## Abstract

The purpose of this paper was to conduct a systematic review and a meta-analysis of studies examining the acute effects of caffeine ingestion on measures of rowing performance. Crossover and placebo-controlled experiments that investigated the effects of caffeine ingestion on measures of rowing performance were included. The PEDro checklist was used to assess the methodological quality of the included studies. Seven studies of good and excellent methodological quality were included. None of the included studies examined on-water rowing. The majority of studies that were included in the meta-analysis used a 2000m rowing distance with only one using 1000m distance. Results of the main meta-analysis indicated that caffeine enhances performance on a rowing ergometer compared to placebo with a mean difference of −4.1 s (95% confidence interval (CI): −6.4, −1.8 s). These values remained consistent in the analysis in which the study that used a 1000m distance was excluded (mean difference: −4.3 s; 95% CI: −6.9, −1.8 s). We also found a significant increase in mean power (mean difference: 5.7 W; 95% CI: 2.1, 9.3 W) and minute ventilation (mean difference: 3.4 L/min; 95% CI: 1.7, 5.1 L/min) following caffeine ingestion. No significant differences between caffeine and placebo were found for the rating of perceived exertion, oxygen consumption, respiratory exchange ratio, and heart rate. This meta-analysis found that acute caffeine ingestion improves 2000m rowing ergometer performance by ~4 s. Our results support the use of caffeine pre-exercise as an ergogenic aid for rowing performance.

## 1. Introduction

Caffeine is one of the most consumed substances in the world [1]. The effects of caffeine on exercise performance have received substantial attention in the literature [1,2,3]. Given its potential as an ergogenic aid, caffeine is also often consumed by athletes. For example, Del Coso et al. [4] investigated the prevalence of caffeine use in athletes since the 2004 removal of caffeine from the World Anti-Doping Agency banned list. Of the analyzed athletes, the authors reported that rowers were among the highest users of caffeine. In a subsequent analysis from 2015, rowers were again found to have very high urine caffeine concentration after competition [5]. 

Even though widely consumed by rowers, there appears to be no scientific consensus as to the effects of caffeine on rowing performance. As an illustration, Skinner et al. [6] reported that, compared to placebo, caffeine ingestion in doses of 2, 4, and 6 mg per kg of body mass, did not improve 2000 m rowing ergometer performance in a group of ten competitive male rowers. These findings are in contrast to those of Bruce et al. [7] who, in a group of eight trained rowers, observed that 6 mg/kg and 9 mg/kg doses of caffeine resulted in an improved 2000 m rowing ergometer performance (i.e., caffeine ingestion reduced the time needed to complete the distance). Discrepancies in these findings are evident even though both studies were conducted in trained rowers and used the same performance tests. 

It is possible that some of the studies examining the effects of caffeine on rowing ergometer performance were statistically underpowered to observe significant effects, resulting in a type II error. The considerable inter-individual variation in responses to caffeine’s effects on exercise performance [8] coupled with underpowered studies might have produced that the potential ergogenic effect of caffeine on rowing is disguised, particularly after the solid evidence of this substance in other sport disciplines [9]. In that regard, meta-analysis presents a method that allows pooling of studies that address a similar research question. As such, a meta-analysis may provide greater confidence in the results given that meta-analytical findings are based on the entire body of evidence, as opposed to those from a single study. A recent meta-analysis of four studies showed that caffeine ingestion, as compared to placebo, improved mean power output during rowing by 2.1% [10]. A limitation of this review is that the meta-analysis was performed on percent changes, which are highly influenced by baseline values, and may, in some cases, even be misleading [11]. In addition, Turner et al. [10] did not analyze the mean differences in time needed to complete the set rowing distance following the ingestion of caffeine and placebo, an outcome that is of interest as well. 

Given the widespread anecdotal use of caffeine in rowers [4,5], and the lack of scientific consensus on the effects of caffeine on rowing performance, this paper aims to conduct a systematic review and a meta-analysis of studies examining the effects of caffeine ingestion on rowing performance. Such an analysis would be of interest to the following: (a) athletes competing in rowing; (b) sports nutritionists; (c) coaches; and (d) researchers interested in further exploring the influence of caffeine supplementation on rowing performance.

## 2. Materials and Methods

### 2.1. Search Strategy

The present review followed the Preferred Reporting Items for Systematic Reviews and Meta-Analyses (PRISMA) guidelines. Articles were identified using the following search strategy: (caffeine OR coffee) AND (rowing OR oarsmen OR oarswomen OR sculls OR ergometer). In total, we searched five different databases, namely, PubMed/MEDLINE, Scopus, Web of Science, SPORTDiscus, and Open Access Theses and Dissertations. The search was conducted without any year restrictions. To avoid publication bias, we examined both peer-reviewed literature as well as unpublished documents such as master’s theses, doctoral dissertations, and conference abstracts. The search was, however, restricted to studies published only in English. Secondary searches consisted of screening the reference lists of the included studies as well as the examination of the papers that have cited the included studies through the Scopus database. To prevent any selection bias, the search for studies was performed by two authors (the first and second author). After conducting the searches, the authors compared the lists of included and excluded studies; if there were any discrepancies in the included studies, the final decision was made through discussion and agreement with a third author (PM). The search was performed on 16 April 2019.

### 2.2. Inclusion Criteria

We included studies that met the following criteria: (a) published in English; (b) investigated the effects of caffeine ingestion in any form (as long as the effect of caffeine could be isolated) on rowing performance (expressed as the time needed to complete a given distance or the total distance covered in a pre-determined amount of time); (c) employed a crossover placebo-controlled design; and (d) included apparently healthy human participants. 

### 2.3. Study Coding and Data Extraction

The following information was extracted on a predefined coding sheet by two authors (the first and second author) of the paper: (a) study design; (b) sample characteristics and their rowing experience; (c) caffeine dose and caffeine form; (d) timing of caffeine ingestion; (e) reported side-effects; (f) rowing conditions (i.e., on-water or laboratory-based tests); and (g) the rowing performance values and the associated physiological responses of the caffeine and placebo conditions. When needed, The Web Plot Digitizer software (V.3.11. Austin, TX, USA: Ankit Rohatgi, 2017) was used to extract data from figures.

### 2.4. Methodological Quality

The PEDro checklist was used to assess the methodological quality of the included studies [12]. The maximal score on the 11-point PEDro scale is ten (the first item does not contribute to the summary score). These 11 items refer to specification of eligibility criteria (item 1), study randomization (item 2), concealed allocation (item 3), similarity of groups at baseline (item 4), blinding (items 5, 6, and 7), number of participants that completed the trials (item 8), intention to treat (item 9), reporting of results (item 10), and reporting of variability in the results (item 11). Based on the summary score, the studies were classified as: (a) excellent quality (9–10 points); (b) good quality (6–8 points); (c) fair quality (4–5 points); or (d) poor quality (less than 3 points), as done in previous reviews [13,14,15]). Two authors (the first and second author) performed the appraisal of methodological quality independently. Any differences in the assessment between the authors were resolved through discussion and agreement.

### 2.5. Statistical Analysis

The extracted data were used to calculate the difference in means and 95% confidence intervals (CIs). The difference in means was calculated using the mean ± standard deviation of the caffeine and placebo conditions, the sample size from each study, and the correlation between the caffeine and placebo conditions. None of the included studies presented correlation values. Therefore, correlation was estimated using the following formula from the Cochrane Handbook:

$$r\;=\;\frac{S_{placebo}^{2}+S_{caffeine}^{2}-S_{D}^{2}}{2\;\cdot \;S_{placebo}\;\cdot \;S_{caffeine}}$$

*S* represents the standard deviation while *S_D_* is the standard deviation of the difference score, calculated as:

$$S_{D\;}\;=\;{\left(\frac{S_{placebo}^{2}}{n}+\frac{S_{caffeine}^{2}}{n}\right)}^{1/2}$$

If studies used multiple doses, we calculated the differences in means and variance for each of the caffeine doses and used the average values for the main analysis. Negative values represent increased performance (i.e., a decrease in the time needed to complete the event). A sensitivity analysis was performed by excluding the study from Duncan [16]. To explore if the change in rowing performance following caffeine ingestion was accompanied with changes in the rating of perceived exertion (RPE), mean power, oxygen consumption (VO_2_), respiratory exchange ratio (RER), minute ventilation (V_E_), and heart rate (HR), additional meta-analyses were performed for these outcomes. Data for all outcomes are reported as mean difference (where the data was expressed in the same units) and standardized mean differences (SMD). 

Heterogeneity was assessed using the *I*^2^ statistic. The following classification was used for heterogeneity: (a) low levels (≤50%); (b) moderate levels (50–75%); and (c) high levels (>75%) of heterogeneity. Publication bias could not be assessed given that there were less than ten included studies. The statistical significance threshold was set at *p* < 0.05. The meta-analysis was conducted using the random-effects model in the Comprehensive Meta-analysis software, version 2 (Biostat Inc., Englewood, NJ, USA). 

## 3. Results

### 3.1. Search Results

**Figure 1:**
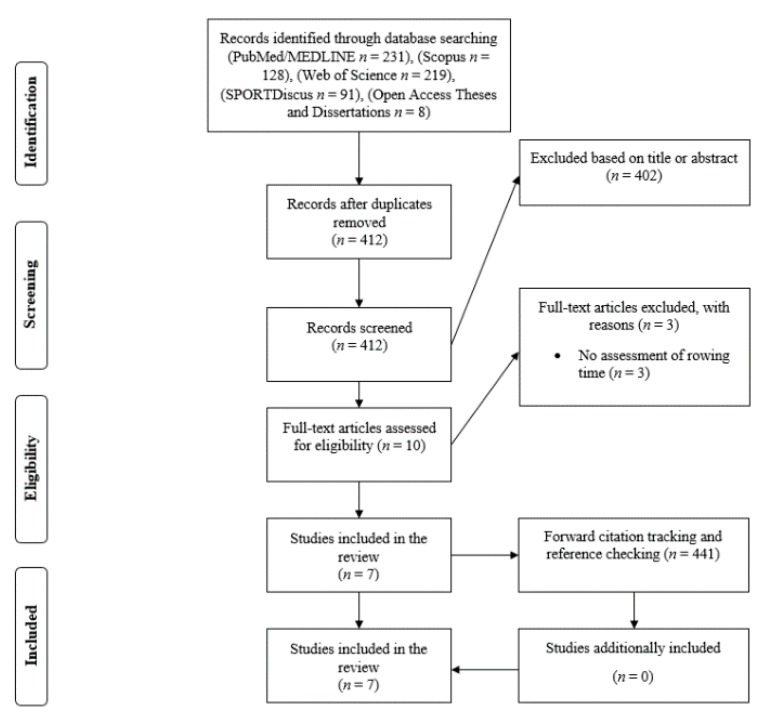
Flow diagram of the search process.

The search through the five databases produced a total of 677 search results. Of that number, ten full-text papers were read. Out of the ten read studies, seven satisfied the inclusion criteria [6,7,16,17,18,19,20]. A total of 441 results appeared in the secondary searches; however, secondary searches did not result in the inclusion of any additional studies. Six studies were published in peer-reviewed journals, while one study was published as a book chapter [14]. The flow diagram of the search is presented in Figure 1.

### 3.2. Study Characteristics

The pooled number of participants across the seven studies is 71 (men *n*: 58, females *n*: 13). In five studies the sample consisted of competitive rowers. Participants in the remaining two studies had previous experience with rowing but were not competitive rowers. All included studies used a Concept II rowing ergometer for the testing sessions. None of the included studies examined the effects of caffeine on on-water rowing performance. Five studies used a double-blind study design, one utilized a single-blind study design, and in one study there was no blinding (Table 1). Most studies used caffeine doses adjusted relative to body weight in a capsule or liquid form; one study used an absolute dose of caffeine (100 mg in the form of a gel). The doses ranged from 1.3 mg/kg to 9 mg/kg. The effectiveness of blinding was assessed in two studies. One study [19] reported that 80% of the total sample was able to correctly identify the caffeine condition. Another study [6] reported that the range of correct identification was from 10% to 50% for the three caffeine doses employed. All but one study [16] reported control of nutritional intake and physical activity on the days before the caffeine and placebo supplementation. The summary of all included studies is presented in Table 1.

### 3.3. Methodological Quality

The average methodological quality score on the PEDro checklist was 9 (range 7 to 10). Based on these scores, five studies were classified as excellent methodological quality while two studies were classified as good methodological quality. The results of the quality assessment of the included studies can be found in Table 2. 

### 3.4. Meta-Analysis Results

**Figure 2:**
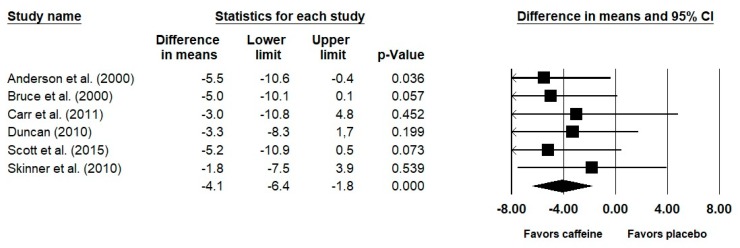
Results of the meta-analysis. Values are expressed as a difference in means and 95% confidence interval (95% CI). The size of the squares reflects the statistical weight of each study. Negative values denote improvements in performance, that is, a reduction in the time needed to complete the rowing distance.

Results of the meta-analysis indicated a significant difference (*p* < 0.001) between the placebo and caffeine conditions in terms of performance on a rowing ergometer (Figure 2). The pooled difference in means favored the caffeine condition and amounted to −4.1 s (95% CI: −6.4, −1.8 s; *I*^2^ = 0%; SMD: 0.41, 95% CI: 0.15, 0.68; *p* = 0.002; *I*^2^ = 0%). These values remained consistent in the sensitivity analysis as the pooled difference in means amounted to −4.3 s (95% CI: −6.9, −1.8 s; *p* < 0.001; *I*^2^ = 0%; SMD: 0.43, 95% CI: 0.13, 0.73; *p* = 0.005; *I*^2^ = 0%). The percent changes in performance following caffeine ingestion ranged from 0.3% to 1.4% (Table 1). The analysis for mean power indicated significant favoring of caffeine, as compared to placebo (expressed as SMD: 0.09; 95% CI: 0.03, 0.15; *p* = 0.004; *I*^2^ = 0%; expressed as mean difference: 5.7 W; 95% CI: 2.1, 9.3 W; *p* = 0.002; *I*^2^ = 0%). 

There was no significant difference between the placebo and caffeine conditions for RPE values (expressed as SMD: 0.40; 95% CI: –0.20, 1.00; *p* = 0.176; *I*^2^ = 49%; expressed as mean difference; −0.30; 95% CI: −0.80, 0.30; *p* = 0.320; *I*^2^ = 0%), VO_2_ (SMD: 0.06; 95% CI: −0.02, 0.15; *p* = 0.119; *I*^2^ = 0%), RER (expressed as SMD: −0.17; 95% CI: −0.50, 0.16; *p* = 0.322; *I*^2^ = 0%; expressed as mean difference: −0.02; 95% CI: −0.05, 0.01; *p* = 0.261; *I*^2^ = 0%), or HR (expressed as SMD: 0.02; 95% CI: −0.14, 0.18; *p* = 0.803; *I*^2^ = 7%; expressed as mean difference: 0.05 beats/min; 95% CI: −1.34, 1.44 beats/min; *p* = 0.940; *I*^2^ = 0%). We found significant increases in V_E_ following the ingestion of caffeine, as compared to placebo (expressed as SMD: 0.25; 95% CI: 0.09, 0.40; *p* = 0.001; *I*^2^ = 0%; expressed as mean difference: 3.4 L/min; 95% CI: 1.7, 5.1 L/min; *p* > 0.001; *I*^2^ = 0%).

## 4. Discussion

The primary finding of this meta-analysis is that caffeine ingestion significantly improves 2000 m rowing ergometer performance by approximately 4 s, as compared to placebo. This improvement in performance was accompanied by a small increase in average power output (~6 W) and V_E_ (~3 L/min). These results, therefore, support the use of caffeine as an ergogenic aid for rowing performance. The included studies were classified as good or excellent methodological quality. As presented in Figure 2, the difference in means in all included studies favored the caffeine condition. This may indeed suggest that some of the individual studies were statistically underpowered to observe significant differences between placebo and caffeine, thus further reinforcing the importance of the results present in this meta-analysis. 

One important consideration is that all the studies cited in this meta-analysis utilized individual time trials on a rowing ergometer to determine the ergogenic effects of caffeine. On-water trials were not used likely given that on-water rowing performance can be affected by environmental conditions. Also, the importance of rowing technique is less evident for ergometer rowing than on-water rowing. On-water rowing is a complex task and comprises components such as balance, economy, and maintenance of boat-speed during the recovery phase, none of which can be measured on an ergometer [21]. To rigorously control for confounding factors, researchers opt to test the acute effects of caffeine ingestion on performance using a rowing ergometer. From a study design perspective, using a rowing ergometer might be considered a methodological strength given its high reliability [22]. However, from a practical standpoint, it may also be viewed as a limitation given that it is unclear to what extent can the effects of caffeine observed on a rowing ergometer be extrapolated to on-water rowing performance. Jürimäe et al. [23] noted a high correlation (*r* = 0.72) between rowing ergometer performance and on-water performance for single sculls and these observations would indicate that our meta-analytical results might also be of value for on-water rowing. This is further supported by the finding that 2000m rowing ergometer performance times exhibit moderate to strong correlations with rankings at the World Rowing Championships in most (albeit not all) rowing events [24]. Therefore, while indicative, caution must be practiced in attempting to extrapolate the results of the individual performance tests utilized in caffeine research to the real-world setting of rowing competitions. 

We did not find significant differences between caffeine and placebo conditions in the majority of the physiological responses that occurred during the rowing task. However, we found a small but significant increase in V_E_ following the ingestion of caffeine as compared to placebo. The increase in V_E_ following caffeine ingestion might not be due to caffeine per se, as it seems more likely this occurred as a consequence of the improvements in performance. This may especially be the fact if we consider that caffeine’s ergogenic effect on exercise performance is mostly due to its ability to bind to adenosine receptors and increase motor unit recruitment [2,25].

Out of the seven included studies, only one [18] including a sample consisting exclusively of women. In all other studies, the researchers either included only men or employed a mixed-sex sample. Therefore, we were not able to explore if the effects of caffeine on rowing performance differ between men and women. The studies by Anderson et al. [18] and Bruce et al. [7] essentially used the same design (i.e., the same timing and dose of caffeine ingestion), with the former including females and the latter males as study participants. The difference in means between the placebo and caffeine conditions in these two studies were almost identical (5.0 and 5.5 s) [7,18], respectively. Therefore, these results tentatively suggest that the response to caffeine is similar in men and women, even though this is a topic that should be directly explored in future research. 

The doses of caffeine provided in the included studies ranged from 1.3 mg/kg to 9 mg/kg. Currently, it remains unclear what the ‘optimal’ dose of caffeine is for enhancing rowing performance. Of the studies that used multiple doses, two found similar improvements in performance following the ingestion of 6 and 9 mg/kg of caffeine [7,18]. In contrast, in one study, none of the three employed doses (i.e., 2, 4, and 6 mg/kg) were ergogenic [6]. These differences in the results are likely because the ‘optimal’ dose of caffeine is highly individual, as shown by studies that plot individual participant responses to varying doses of caffeine [26,27]. Therefore, while our analysis reports that caffeine is ergogenic for rowing performance (when considering average responses), the optimal dose and protocol of caffeine supplementation need to be established on a case-by-case basis.

Based on the PEDro checklist, all included studies are classified as good or excellent methodological quality. However, two areas of study design need to be highlighted for future research. The double-blind study design is considered the ‘gold standard’ in the sports nutrition area of research. Five studies did indeed employ such a design; however, one study also used a single-blind design, and in one study, no blinding was used (Table 1). For future investigations, double-blind study designs should be used to even further improve the methodological quality. As noted previously, only two studies explored the effectiveness of the blinding to the caffeine and placebo conditions. Recently, Saunders et al. [28] presented data that suggest that correct supplement identification may influence the outcome of a given exercise task and, therefore, could be a source of bias in the sports supplement line of research. Given these results, we would like to highlight to researchers examining the effects of caffeine on exercise performance to assess the effectiveness of the blinding. Preferably, this assessment should be done both pre- and post-exercise, given that the opinion and response might change from pre- to post-exercise [28].

On a final note, we would like to point out that the participants in the included studies were competitive rowers (or, to a lesser extent, individuals with some experience with rowing). However, the included studies did not involve elite rowing athletes. Therefore, while our results clearly indicate that caffeine may be ergogenic for performance on a rowing ergometer, future studies are needed to explore this topic in elite athletes. 

## 5. Conclusions

Acute caffeine ingestion (as compared to placebo) may improve 2000m rowing ergometer performance in competitive rowers by approximately 4 s. This improvement in performance was accompanied by small increases in power output and V_E_. Our results support the use of acute caffeine supplementation for enhancement in performance on a rowing ergometer. Future studies should explore the optimal dosage of caffeine for maximizing these ergogenic effects as well as attempt to involve rowers of the international/elite rank.

## Figures and Tables

**Table 1:** Summary of the included studies.

Reference	Study Design	Sample	Caffeine Dose	Caffeine Form	Timing of Caffeine Ingestion	Rowing Distance or Minutes of Rowing	Rowing Conditions	Time to Complete the Distance (Placebo Condition—Seconds)	Percent Change *	Reported Side-Effects from Caffeine
Anderson et al. (2000)	Randomized double-blind crossover	8 competitive oarswomen (age: 22 ± 3 years; body mass: 64 ± 4 kg)	6 and 9 mg/kg	Capsule	60 min pre-exercise	2000 m	Concept II rowing ergometer	479 ± 15	6 mg/kg: ↑ 0.7% 9 mg/kg: ↑ 1.4%	None reported.
Bruce et al. (2000)	Randomized double-blind crossover	8 competitive oarsmen **	6 and 9 mg/kg	Capsule	60 min pre-exercise	2000 m	Concept II rowing ergometer	414 ± 15	6 mg/kg: ↑ 1.3% 9 mg/kg: ↑ 1.1%	None reported.
Carr et al. (2011)	Double-blind crossover	8 competitive rowers (6 men and 2 women) (body mass for men: 82 ± 12 kg; body mass for women: 78 ± 6 kg) **	6 mg/kg	Capsule	30 min pre-exercise	2000 m	Concept II rowing ergometer	403.8 ± 23.4	↑ 0.7%	Irregular heartbeat, increased alertness, hand tremor, and feeling hyperactive.
Christensen et al. (2014)	Double-blind crossover	14 competitive rowers (11 men and 1 women) (age: 25 to 27 years; body mass for men: 92 ± 3 kg, or 75 ± 3 kg; body mass for women: 63 kg)	3 mg/kg	Capsule	45 min pre-exercise	6 min rowing	Concept II rowing ergometer	n/a	↑ 0.7%	None reported.
Duncan (2000)	Crossover	12 individuals with some experience in rowing (10 men and 2 women) (age: 22 ± 3 years) **	5 mg/kg	Liquid	60 min pre-exercise	1000 m	Concept II rowing ergometer	231.7 ± 22.6	↑ 1.4%	None reported.
Scott et al. (2015)	Randomized single-blind crossover	13 men with some experience in rowing (age: 21 ± 2 years; body mass: 78 ± 9 kg)	100 mg	Gel	10 min pre-exercise	2000 m	Concept II rowing ergometer	471.4 ± 28.5	↑ 1.1%	None reported.
Skinner et al. (2010)	Randomized double-blind crossover	10 competitive oarsmen (age: 21 ± 1 years; body mass: 88 ± 11 kg)	2, 4 and 6 mg/kg	Capsule	60 min pre-exercise	2000 m	Concept II rowing ergometer	403.8 ± 21	2 mg/kg: ↑ 0.3% 4 mg/kg: ↑ 0.7% 6 mg/kg: ↑ 0.3%	Increased alertness, difficulty sleeping, and hand tremors.

* percent change with caffeine ingestion compared to placebo; ** age or body mass was not reported. ↑ increased performance (i.e., a reduced time to complete the rowing distance or increased rowing distance) with caffeine ingestion as compared to placebo. Data are presented as mean ± SD.

**Table 2:** Results of PEDro checklist quality assessment.

Reference	Item 1	Item 2	Item 3	Item 4	Item 5	Item 6	Item 7	Item 8	Item 9	Item 10	Item 11	Total Score
Anderson et al. (2000)	No	Yes	Yes	Yes	Yes	Yes	Yes	Yes	Yes	Yes	Yes	10
Bruce et al. (2000)	No	Yes	Yes	Yes	Yes	Yes	Yes	Yes	Yes	Yes	Yes	10
Carr et al. (2011)	No	No	Yes	Yes	Yes	Yes	Yes	Yes	Yes	Yes	Yes	9
Christensen et al. (2014)	No	Yes	Yes	Yes	Yes	Yes	Yes	Yes	Yes	Yes	Yes	10
Duncan (2000)	No	Yes	Yes	Yes	No	No	No	Yes	Yes	Yes	Yes	7
Scott et al. (2015)	No	No	No	Yes	No	Yes	Yes	Yes	Yes	Yes	Yes	7
Skinner et al. (2010)	No	Yes	Yes	Yes	Yes	Yes	Yes	Yes	Yes	Yes	Yes	10

Yes = criterion is satisfied; No = criterion is not satisfied.
